# Association of a history of depression with infertility, miscarriage and stillbirth: a longitudinal cohort study

**DOI:** 10.1017/S2045796024000374

**Published:** 2024-11-05

**Authors:** Chen Liang, Hsin-Fang Chung, Annette J. Dobson, Gita D. Mishra

**Affiliations:** School of Public Health, The University of Queensland, Herston, QLD, Australia

**Keywords:** depression, infertility, miscarriage, stillbirth, longitudinal study

## Abstract

**Aims:**

The role of depression in subsequent infertility, miscarriage and stillbirth remains unclear. This study aimed to examine the association of a history of depression with these adverse outcomes using a longitudinal cohort study of women across their reproductive life span.

**Methods:**

This study used data from participants in the Australian Longitudinal Study on Women’s Health who were born in 1973–1978. Participants (*N* = 8707) were followed up every 3 years from 2000 (aged 22–27) to 2018 (aged 40–45). Information on a diagnosis of depression was collected from each survey, and antidepressant medication use was identified through pharmaceutical prescription data. Histories of infertility, miscarriage, and stillbirth were self-reported at each survey. Time-lagged log-binomial models with generalized estimating equations were used to assess the association of a history of depression up to and including in a given survey with the risk of fertility issues in the next survey.

**Results:**

Women with a history of depression (excluding postnatal depression) were at higher risk of infertility [risk ratio (RR) = 1.34, 95% confidence interval (CI): 1.21–1.48], miscarriage (RR = 1.22, 95%CI: 1.10–1.34) and recurrent miscarriages (≥2; RR = 1.39, 95%CI: 1.17–1.64), compared to women without a history of depression. There were too few stillbirths to provide clear evidence of an association. Antidepressant medication use did not affect the observed associations. Estimated RRs of depression with infertility and miscarriage increased with age.

**Conclusions:**

A history of depression was associated with higher risk of subsequent infertility, miscarriage and recurrent miscarriages.

## Introduction

Depression is a common and serious mood disorder, which can negatively affect one’s feelings, thoughts and daily functioning. Depression is more common in females than males (Global Burden Disease 2019 Mental Disorders Collaborators, 2023). Globally, the prevalence of depression (major depressive disorder) is around 3.1% among women and 1.9% among men, affecting over 115 million women and 69 million men (Institute of Health Metrics and Evaluation, 2023). Infertility, miscarriage and stillbirth are common fertility issues. Around 12.4% of women at reproductive age experience infertility, 15.3% of all recognised pregnancies result in miscarriage and 1.8% of total births end in stillbirth (Lawn *et al.* 2016; Mascarenhas *et al.* 2012; Quenby *et al.*, 2021). Infertility is a condition characterized by failing to establish a clinical pregnancy after 12 months of regular, unprotected sexual intercourse or due to an impairment of a person’s capacity to reproduce, either as an individual or with his/her partner (Zegers-Hochschild *et al.*, 2017). Both miscarriage and stillbirth describe the loss of a pregnancy. Miscarriage is the spontaneous loss of an intrauterine pregnancy prior to 20 completed weeks of gestational age, and stillbirth is the death of the foetus after 20 completed weeks of gestational age (Centers for Diseases Control and Prevention, 2024).

Infertility, miscarriage and stillbirth, as life crises, have been reported to be associated with subsequent depression (Farren *et al.*, 2016, 2020; Kiani *et al.*, 2021; Rooney and Domar 2018). However, previous studies on pre-existing depression and subsequent fertility issues are limited. These conditions could be linked through endocrine changes (e.g., low oestrogen and underactive thyroid) and shared risk factors (e.g., underweight, obesity and smoking) (Chaiton *et al.* 2009; Jung *et al.* 2017; Tichomirowa *et al.* 2005). As far as we know, only five previous studies have examined the association between pre-existing depression and later risk of fertility issues (Jimenez-Solem *et al.*, 2013; Lapane *et al.* 1995; Magnus *et al.* 2021; Sugiura-Ogasawara *et al.*, 2002; Wang *et al.*, 2021). These studies, however, have some notable limitations, such as small sample size, retrospective data collection and inconsistent ascertainment of depression. Different study populations (e.g., pregnant women, pregnant women who have previously experienced one miscarriage and pregnant women who have experienced two consecutive miscarriages) make it difficult to compare their results. Therefore, the role of a history of depression in the subsequent experience of infertility, miscarriage and stillbirth remains unclear. In addition, no previous studies have compared the effect of age on the association between a history of depression and these three fertility issues. It is known that the risk of infertility, miscarriage and stillbirth goes up after age 35 possibly due to the decrease in egg quality and higher risk of chronic conditions (Lawn *et al.*, 2016; Mascarenhas *et al.*, 2012; Quenby *et al.*, 2021). The effects of depression acting through the associated endocrine changes (e.g., low oestrogen and underactive thyroid) or shared risk factors (e.g., obesity and smoking) may accumulate with age (Chaiton *et al.*, 2009; Jung *et al.*, 2017; Tichomirowa *et al.*, 2005).

Therefore, this study used data from women born in 1973–1978 who have participated in the Australian Longitudinal Study on Women’s Health (ALSWH) to test the hypotheses that: (1) women with a history of depression are at a higher risk of subsequent infertility, miscarriage or stillbirth and (2) the strength of these associations may increase as women get older.

**Figure 1.**
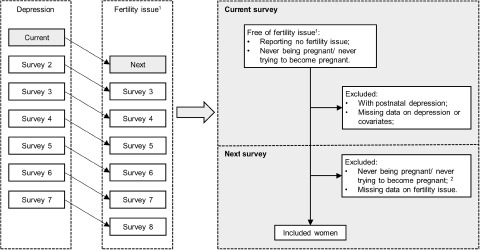
One-survey lagged data structure with inclusion and exclusion criteria.

^1^Fertility issue: infertility, miscarriage, recurrent miscarriages or stillbirth. When the fertility issue of interest was miscarriage or stillbirth, women reporting not having the fertility issue or never being pregnant in the current survey were included. When the fertility issue of interest was infertility, women reporting no infertility or never trying to be pregnant in the current survey were included.

^2^When the fertility issue of interest was infertility, women reporting never trying to be pregnant in the next survey were excluded. When the fertility issue of interest was miscarriage or stillbirth, women reporting never being pregnant in the next survey were excluded.

## Methods

### Study design and participants

The ALSWH is a national study that commenced in 1996 with three age cohorts of women randomly selected from the database of Medicare, the universal national health insurance system, which covers all Australian citizens and permanent residents. Further details on the study design and methods have been provided previously (Dobson *et al.* 2015; Lee *et al.*, 2005). The present study used data from the women in the 1973–1978 cohort who completed survey 2 in 2000 when they were aged 22–27 years and who did not report any fertility issues up to that time. Survey 2 was set as the analytic baseline, as this was when information on depression was firstly collected. The analysis then used longitudinal data from repeated surveys, conducted at 3 yearly intervals, until survey 8 in 2018 when the women were aged 40–45 years. It also included record linkage data for the same period from the Pharmaceutical Benefits Scheme (PBS), which records all prescriptions filled for government subsidised medicines in Australia.

**Figure 2.**
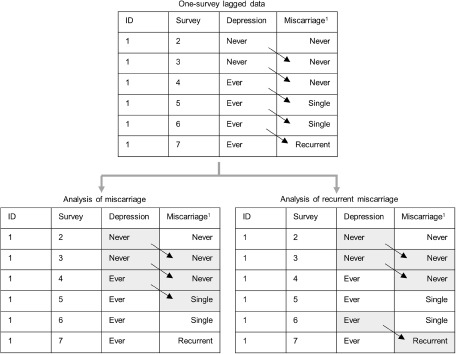
Example of one-survey lagged data contributing to the analyses of miscarriage and recurrent miscarriages.

^1^History of miscarriage up to each survey.

To explore the time-lagged effect of a history of depression up to the current survey on the risk of adverse pregnancy outcome (specifically miscarriage, recurrent miscarriages or stillbirth) in the next survey, women meeting the following criteria were included (Fig. 1): (1) no reported history of the adverse pregnancy outcome of interest up to the current survey; (2) no reported postnatal depression up to the current survey; (3) reported ever being pregnant in the next survey; (4) no missing data on the exposure, outcome or covariates. Women with postnatal depression were excluded because rapid reproductive hormone changes (e.g., oestrogen and progesterone) during pregnancy and immediately after delivery are etiologically important in the development of postnatal depression, which is different from non-postnatal depression (Stewart and Vigod 2019). In the analysis of recurrent miscarriage, records with a history of single miscarriage in the next survey were excluded, because for these records the association between depression and lagged miscarriage status could be bidirectional (Fig. 2). For the analysis of infertility, women were included if they met the following criteria: (1) no reported infertility up to the current survey; (2) no reported postnatal depression up to the current survey; (3) at the next survey reported ever trying to become pregnant; (4) no missing data on the exposure, outcome or covariates.

Highlighted records contribute to the corresponding analysis. In the analysis of miscarriage, followed-up data until the occurrence of miscarriage were used. In this example, survey 2→3, 3 →4 and 4 →5 contribute to the analysis of miscarriage. In the analysis of recurrent miscarriages, follow-up data until the occurrence of recurrent miscarriages were used, and one-survey lagged records with a history of single miscarriage in the next survey were excluded. In this example, survey 2→3, 3→4 and 6→7 contribute to the analysis of recurrent miscarriages.

### Depression

Women were asked whether they had been diagnosed with depression (but not postnatal depression) in survey 2–8, or received antidepressant medications (e.g., Prozac and Aropax) in surveys 2 and 3 only. In PBS data (July 2003–December 2018), antidepressant medications were identified by the Anatomical Therapeutic Chemical codes N06AA, N06AB, N06AF, N06AG, N06AX03, N06AX05, N06AX06, N06AX07, N06AX09-N06AX14, N06AX16, N06AX18, N06AX21-N06AX24, N06AX26 and N06CA. Women were considered to have a history of depression in one survey, if they reported a diagnosis of depression or use of antidepressant medications in that survey, or had records in the PBS data of two or more filled prescriptions for antidepressants within a single year during the preceding interval between surveys. If a woman was identified with depression in one survey, her history of depression would be carried forward to subsequent surveys. Similarly, the history of antidepressant medication use at each survey was created. The use of antidepressant medication supports the validity of self-reported diagnosis of depression and may indicate more severe depression. To explore whether the risk of fertility issues differed by the use of antidepressant medications, women were also categorized into three groups based on their histories of depression and antidepressant medication use.

History of postnatal depression was collected at each survey. Women who reported doctor diagnosed postnatal depression or deliveries associated with postnatal depression were considered to have postnatal depression. In each time-lag (current survey → next survey), women who had ever reported postnatal depression up to the current survey were excluded.

### Infertility, miscarriage and stillbirth

In each survey, self-reported data on infertility, miscarriage and stillbirth during the preceding survey interval were obtained. Women who had tried unsuccessfully to become pregnant for 12 months or more were considered to have infertility and women who had never tried to become pregnant were also identified and excluded from the analysis of infertility. The number of miscarriages was collected. Recurrent miscarriages were defined as two or more miscarriages, which could be interspersed by live birth(s) (Quenby *et al.*, 2021).

### Covariates

Covariates were self-reported at each survey, including gravidity (never and ever been pregnant), education level (did not complete high school, completed high school, trade or certificate and university or higher level), marital status (married/de facto, separated/divorced/widowed and single), body-mass index (BMI), smoking status (never, past and current smoker) and alcohol intake (never drink, rarely drink ≤14, 15–28, and ≥29 drinks per week). BMI was categorized as underweight, normal weight, overweight and obese (<18.5, 18.5–22.9, 23–27.4 and ≥27.5 kg/m^2^ for Asian women; <18.5, 18.5–24.9, 25.0–29.9 and ≥30.0 kg/m^2^ for other women) (Expert Penal on the Identification, 1998; WHO Expert Consultation, 2004). Above covariates except gravidity were considered as confounding factors (Chaiton *et al.*, 2009; Jung *et al.*, 2017; Kessler and Bromet 2013; Lawn *et al.*, 2016; Nunes 2023; Quenby *et al.*, 2021; Vander Borght and Wyns 2018), among which BMI, smoking status and alcohol intake might also act as mediators (Boden and Fergusson 2011; Chaiton *et al.*, 2009; Konttinen *et al.*, 2019). In the analysis, categories of underweight and normal weight were combined owing to the small number of participants in the underweight group. Similarly, alcohol consumption categories of ≤14, 15–28 and ≥29 drinks per week were combined.

### Statistical analyses

Continuous variables were summarized by the median and quartiles, and categorical variables by the number and percentage. Binomial models with a log link function were used to estimate the risk ratios (RRs) and two-sided 95% confidence intervals (CIs) for the association of a history of depression (never and ever) in the current survey with the risk of fertility issues (e.g., infertility, miscarriage, recurrent miscarriages and stillbirth) in the next survey. Generalized estimating equation models were used to take account of the repeated measures in the longitudinal data from six surveys. An exchangeable correlation structure was used. Models were firstly adjusted for education level and marital status (model 1), and additionally adjusted for BMI, smoking status and alcohol intake in the current survey (model 2). Time trend was taken into account by including a fixed effect of survey number (and hence age). Secondly, associations were assessed for depressed women with and without a history of antidepressant medication use. Women were reclassified into three groups: women without depression, women with depression without antidepressant use and women with depression and antidepressant use. The average RR across all surveys was assessed through model without an interaction term between history of depression and survey number. To explore how the strength of association changed with age, models with an interaction term (depression * survey number) were used. However, this approach was not applied to stillbirth due to the small number of stillbirths in each survey.

Several sensitivity analyses were conducted. First, models were additionally adjusted for a self-reported history of anxiety, because anxiety and depression are highly comorbid with each other (Kalin 2020). Second, analyses were restricted to records in which women reported no history of any fertility issues up to the current survey, because pre-existing infertility or pregnancy loss can be associated with a higher risk of depressive symptoms and a higher risk of subsequent miscarriage or stillbirth (Agenor and Bhattacharya, 2015). Third, analyses were restricted to records of women who had children in the current survey to exclude the potential influence of nulliparity. All statistical analyses were performed using SAS version 9.4 (SAS Institute Inc, Cary, North Carolina, USA; procedure GENMOD for generalized estimating equation time-lagged models).

## Results

Overall, 8707 women from the 1973–1978 ALSWH cohort were included in this study, of whom 7866, 6867 and 7337 participants contributed to the analysis of infertility, miscarriage and stillbirth respectively due to different inclusion/exclusion criteria (Figures S1–S3). At the analytic baseline, their median (quartiles) age was 24.6 (23.3, 25.9) years and they were followed up until a median (quartiles) age of 41.7 (40.0, 43.0) years. By the end of the follow-up period, 3136 (36.0%) women had at least one record of depression (physician-diagnosed or medication). Table 1 shows the baseline characteristics of women with and without any record of depression, and women with depression were more likely to be less educated, have obesity and to be a past or current smoker. During the follow-up period, 2146 (27.3%), 2277 (33.2%) and 278 (3.8%) women experienced infertility, miscarriage and stillbirth respectively, while 729 (10.6%) experienced recurrent miscarriages (two or more miscarriages). In the analysis of infertility, miscarriage and stillbirth, characteristics of women who were included or excluded due to missing data were compared. The excluded women were likely to be less educated, and more likely to be current smokers (Table S1–S3).

**Table 1.** Baseline characteristics of women with and without any record of diagnosis or treatment for depression over the study period

	Depression			
	Never (*N* = 5,571)		Ever (*N* = 3,136)	
	*N*	%	*N*	%
Race				
White	5,385	96.7	3,090	98.5
Asian	123	2.2	26	0.8
Other	63	1.1	20	0.6
Education level				
Did not complete high school	462	8.3	375	12.0
Completed high school	1,481	26.6	866	27.6
Trade or diploma	1,394	25.0	841	26.8
University or higher degree	2,229	40.1	1,053	33.6
Marital status				
Married/de facto	2,237	47.6	1,284	47.2
Divorced/separated/widowed	42	0.9	52	1.9
Single	2,426	51.6	1,382	50.9
Body-mass index (kg/m^2^)*				
<18.5	263	5.9	175	6.7
18.5–24.9	2,881	64.5	1,547	59.5
25.0–29.9	938	21.0	530	20.4
≥30.0	388	8.7	348	13.4
Alcohol intake by NHMRC (dinks per week)				
Non-drinker	379	8.1	221	8.2
Rarely drink	1,287	27.4	775	28.6
≤14	2,898	61.7	1,589	58.6
15–28	131	2.8	112	4.1
≥29	6	0.1	15	0.6
Smoking status				
Never smoker	2,923	62.2	1,375	50.9
Past smoker	657	14.0	438	16.2
Current smoker	1,118	23.8	891	33.0

* For Asian women, body-mass index was categorized as <18.5, 18.5–22.9, 23.0–27.4, and ≥27.5 kg/m^2^.

### Infertility

**Figure 3.**
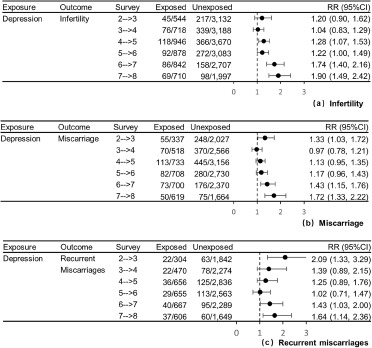
The associations of history of depression with the risk of subsequent infertility, miscarriage and recurrent miscarriages by survey. (a) infertility, (b) miscarriage and (c) recurrent miscarriages.

*Note:* Models were adjusted for education level (did not complete high school, completed high school, trade or certificate and university or higher degree), marital status (married/de facto, divorced/separated/widowed and single), body-mass index (<18.5, 18.5–22.9, 23.0–27.4 and ≥27.5 kg/m^2^ for Asian women; <18.5, 18.5–24.9, 25.0–29.9 and ≥30.0 kg/m^2^ for other women), smoking status (never smoker, past smoker and current smoker) and alcohol intake [never drink, rarely drink and drinker (i.e., ≤14, 15–28 and ≥29 drinks per week)]. The time trend and subject effect were taken into account by including a fixed effect of survey number and a clustering effect for subject. An interaction term between the exposure and survey number was included. In the analyses of infertility, *p*-value for the interaction term depression * survey was 0.0033. In the analyses of miscarriage, *p*-value for the interaction term depression * survey was <0.0001. In the analyses of recurrent miscarriages, *p*-value for the interaction term depression * survey was 0.2616.

Among women who had ever tried to become pregnant, women with a history of depression were at 34% higher risk of infertility in the next survey (RR = 1.34, 95%CI: 1.21–1.48, Table 2) compared to women without a history of depression after adjusting for covariates (i.e., education level, marital status, BMI, smoking status and alcohol intake). The association between a history of depression and infertility differed across survey (*p* = 0.003), exhibiting an upward trend in estimated RRs as the women became older (Fig. 3A). Higher RRs were observed in the last two surveys (RR = 1.74, 95%CI: 1.40–2.16 and RR = 1.90, 95%CI: 1.49–2.42), when women were aged 39.6 (38.4, 40.9) and 42.0 (41.0, 44.0) years respectively. The data did not show a significant difference in the strength of associations between women receiving antidepressant medication or not as their 95%CIs overlapped (Table 2, Figure S4A).

**Table 2.** The association of history of depression (including mediation), history of depression diagnosis with and without antidepressant medication with the risk of subsequent infertility, miscarriage, and stillbirth

Exposure		Crude model	Adjusted model 1	Adjusted model 2
Outcome = infertility				
Depression	Never	Ref.	Ref.	Ref.
	Ever	1.28 (1.17, 1.42)	1.34 (1.21, 1.48)	1.34 (1.21, 1.48)
Depression	Never & never	Ref.	Ref.	Ref.
Diagnosis	Ever & never	1.24 (1.10, 1.39)	1.29 (1.14, 1.45)	1.28 (1.14, 1.44)
Medication	Ever & ever	1.36 (1.18, 1.56)	1.44 (1.25, 1.66)	1.43 (1.24, 1.65)
Outcome = miscarriage				
Depression	Never	Ref.	Ref.	Ref.
	Ever	1.16 (1.06, 1.28)	1.21 (1.09, 1.33)	1.22 (1.10, 1.34)
Depression	Never & never	Ref.	Ref.	Ref.
Diagnosis	Ever & never	1.18 (1.05, 1.33)	1.22 (1.08, 1.37)	1.23 (1.09, 1.38)
Medication	Ever & ever	1.14 (0.98, 1.31)	1.19 (1.03, 1.38)	1.20 (1.04, 1.39)
Outcome = recurrent miscarriage				
Depression	Never	Ref.	Ref.	Ref.
	Ever	1.37 (1.16, 1.62)	1.45 (1.22, 1.71)	1.39 (1.17, 1.64)
Depression	Never & never	Ref.	Ref.	Ref.
Diagnosis	Ever & never	1.38 (1.13, 1.69)	1.44 (1.18, 1.77)	1.40 (1.14, 1.71)
Medication	Ever & ever	1.35 (1.07, 1.72)	1.45 (1.14, 1.84)	1.37 (1.08, 1.75)
Outcome = stillbirth				
Depression	Never	Ref.	Ref.	Ref.
	Ever	1.05 (0.77, 1.43)	0.99 (0.72, 1.35)	0.98 (0.71, 1.34)
Depression	Never & never	Ref.	Ref.	Ref.
Diagnosis	Ever & never	0.94 (0.63, 1.38)	0.89 (0.60, 1.31)	0.88 (0.59, 1.30)
Medication	Ever & ever	1.25 (0.81, 1.94)	1.15 (0.74, 1.80)	1.15 (0.73, 1.80)

*Note*: The crude model took time (age) trend and subject effect into account by including a fixed effect of survey number and a clustering effect for subject. Adjusted model 1 was adjusted for education level (did not complete high school, completed high school, trade or certificate and university or higher degree) and marital status (married/de facto, divorced/separated/widowed and single). Adjusted model 2 was additionally adjusted for body-mass index (<18.5, 18.5–22.9, 23.0–27.4, and ≥27.5 kg/m^2^ for Asian women; <18.5, 18.5–24.9, 25.0–29.9, and ≥30.0 kg/m^2^ for other women), smoking status (never smoker, past smoker and current smoker) and alcohol intake [non-drink, rarely drink and other (i.e., ≤14, 15–28 and ≥29 drinks per week)]. No interaction term was included in any of the above models.

### Miscarriage

Among women who had ever been pregnant, the adjusted models showed women with a history of depression were at 22% higher risk of miscarriage (RR = 1.22, 95%CI: 1.10–1.34), and 39% higher risk of recurrent miscarriage (RR = 1.39, 95%CI: 1.17–1.64) in the next survey (Table 2). There was evidence of an interaction effect between a history of depression and age on the risk of miscarriage (*p* < 0.0001), and higher RRs were observed in the first survey (RR = 1.33, 95%CI: 1.03–1.72) and last two surveys (RR = 1.43, 95%CI: 1.15–1.76 and RR = 1.72, 95%CI: 1.33–2.22; Fig. 3B). The analysis of recurrent miscarriages also indicated higher RRs in the first and last two surveys, even though the CIs were wide and overlapped (Fig. 3C). The strengths of associations were similar between depressed women with and without antidepressant use (Table 2, Figure S4B–4C).

### Stillbirth

Due to the small numbers of stillbirth, and hence the wide CIs, the data did not provide clear evidence on the risk of stillbirth among women with a history of depression (Table 2).

### Sensitivity analyses

Additional adjustment of anxiety gave similar results to the main analysis (Table S4). Restricting the analysis to records for women without any fertility issues reported up to the current survey did not change the findings (Table S5). After restricting the analysis to women with children at the current survey, the association of a history of depression with infertility persisted, while the estimated RRs of depression with miscarriage decreased and the CIs became wider (Table S6).

## Discussion

In this prospective national cohort study of 8707 women, a history of a diagnosis of depression was associated with higher risk of subsequent infertility, miscarriage and recurrent miscarriage, and the associations did not differ by the history of antidepressant medication use. Estimated RRs between a history of depression and subsequent infertility increased with age, especially in women’s late 30s and early 40s, while estimated RRs between a history of depression and miscarriage showed a possibly U-shaped pattern with higher RRs in women’s early 20s, late 30s and early 40s. There was no clear evidence of an association between depression and stillbirth.

Similar to the present study, Bagade et al. used ALSWH data to investigate the association of any psychological distress [defined as self-reported diagnosis of anxiety or depression, the 10-item Center for Epidemiological Studies Depression Scale ≥10, the 5-item Mental Health Inventory ≤52 or the Goldberg Anxiety and Depression Scale >5] with infertility (primary infertility: OR = 1.24 95%CI: 1.06–1.45; secondary infertility: OR = 1.27 95%CI: 1.10–1.46) (Bagade *et al.*, 2022). However, Bagade et al. assessed fertility and psychological distress at the same survey, and so they could not examine the direction of associations. In the present study, time-lagged models were used to estimate the association of a history of depression with subsequent fertility issues. As far as we know, there was only one previous study assessing the association of pre-existing depression or depressive symptoms with infertility. Lapane et al.’s study enrolled 339 women, and found some evidence that a history of depressive symptoms, identified from the question ‘have you ever felt so sad, discouraged, or hopeless, or had so many problems that you wondered if anything was worthwhile’, was associated with a subsequent risk of infertility (OR = 1.7, 90%CI: 0.9–3.2) (Lapane *et al.*, 1995). Our data revealed the association between a history of depression and infertility, and the strength of association was similar for depressed women with and without antidepressant medication use.

With regards to miscarriage, Magnus et al. identified 593,009 pregnant women in Norway, and showed that the risk of miscarriage was higher among women with pre-existing depressive disorders (OR = 1.25, 95%CI: 1.23–1.27), but they did not take psychoactive medication use into account (Magnus *et al.*, 2021). In the present study, a history of depression was associated with higher risk of miscarriage (RR = 1.20 95%CI: 1.09–1.32), and the strength of association was similar for depressed women who had never used antidepressants and depressed women who had ever used antidepressants. In addition, our data indicated a stronger association for recurrent miscarriages. Consistent with the present study, two previous studies observed the associations between depressive symptoms and subsequent miscarriage (Sugiura-Ogasawara *et al.*, 2002; Wang *et al.*, 2021). Wang et al.’s study included 2558 Chinese women with one miscarriage, and used the Self-rating Depression Scale to measure depressive symptoms (Wang *et al.*, 2021). They found women with mild (score 53–62), moderate (score 63–72) and severe (score ≥ 72) depression after the first miscarriage were at 29% (OR = 1.29 95%CI: 1.13–4.65), 64% (OR = 1.64 95%CI: 1.02–3.35) and 40% (OR = 1.40 95%CI: 1.02–2.92) higher risk of another miscarriage in the following 2 years (Wang *et al.*, 2021). Another study by Sugiura-Ogasawara et al. enrolled 61 pregnant women with a history of two consecutive first-trimester miscarriages, and used the Symptom Checklist-90 Revised to measure depressive symptoms before the next pregnancy (Sugiura-Ogasawara *et al.*, 2002). This study showed higher symptom scores among women with subsequent miscarriage [(1.04 ± 0.32) vs (0.46 ± 0.50), *P* = 0.04] (Sugiura-Ogasawara *et al.*, 2002). As for stillbirth, one Danish cohort study (*N* = 920,620) assessed the risk of stillbirth after antidepressant (selective serotonin reuptake inhibitor) use during pregnancy, and no association was observed (Jimenez-Solem *et al.*, 2013). In the present study, the small number of stillbirths limited the power to detect any association of a history of depression with stillbirth.

Additionally, the present study explored whether the strength of association changed by age. The ALSWH participants born in 1973–78 were followed up in 3-yearly waves from age 18–23 to age 40–45. The data showed an upward trend in the estimated RRs for infertility, especially in the last two surveys, indicating higher risk of infertility associated with a history of depression in women’s late 30s and early 40s. With regard to miscarriage and recurrent miscarriages, a U-shaped pattern was observed in the estimated RRs. These results indicated a higher risk of subsequent miscarriage or recurrent miscarriages was associated with a history of depression in women’s early 20s, late 30s and early 40s. As far as we know, this is the first study assessing how the association of pre-existing depression with infertility and miscarriage changed with women’s age. However, owing to the sample size, the 95%CIs of RRs by survey were relatively wide, especially for recurrent miscarriages.

Women with depression may experience hypothalamic–pituitary–gonadal axis suppression, which results in decreased levels of oestradiol (Tichomirowa *et al.*, 2005). Low oestradiol level may enhance negative information appraisal and reduced emotional regulation, leading to more negative emotional memory and negative mood state (Tichomirowa *et al.*, 2005). Meanwhile, oestrogen deficiency is a common cause of infertility and miscarriage (Albert and Newhouse 2019). Oestrogen levels originating from the ovaries peak in women’s later 20s, and then decrease slowly before a sharp decrease at the menopause transition. Low oestradiol levels and age-related oestrogen decrease may put depressed women at a higher risk of fertility issues (e.g., infertility and miscarriage), especially when they are in their late 30s and early 40s. In addition, women with depression frequently have significant changes in their hypothalamic–pituitary–thyroid axis, particular reduced thyroid-stimulating hormone followed by a different grade of subclinical hypothyroidism (Tichomirowa *et al.*, 2005). Thyroid hormone plays a critical role in puberty onset, menstrual regulation, ovulation, implantation and embryo development (Dosiou 2020; Krassas *et al.* 2010). Given the multiple effects of thyroid hormones throughout a woman’s reproductive life, low levels of thyroid hormone may impair fertility and increase the risk of miscarriage in both earlier and later life (Dosiou, 2020; Krassas *et al.*, 2010). Besides, women with a history of depression are more likely to experience sexual dysfunction, such as decreased sexual desire, less frequent sexual arousal, and less sexual satisfaction and unplanned or poorly timed pregnancy (Gariepy *et al.* 2016; Laurent and Simons 2009). These negative effects may contribute to fertility challenges or adverse pregnancy outcomes. Other shared risk factors, such as BMI, smoking, and alcohol consumption, may play a role (Chaiton *et al.*, 2009; Jung *et al.*, 2017; Nunes, 2023). However, in the present study, the associations of depression with infertility and miscarriage did not change after additional adjustment for these factors.

In the present study, follow-up data from women’s early 20s to early 40s enable researchers to assess the relationship between a history of depression and later risk of fertility issues. However, several limitations need to be acknowledged. Women with depression might be unidentified or identified later, so that a depression diagnosis or use of antidepressant medication might not be reported or reported later. Besides, the effects of different types or timing of antidepressant medication were not assessed due to data limitations. The data on government subsidised medication, including type and date, were incomplete before April 2012 (around survey 4) (Kemp *et al.* 2023). In the 3-yearly survey data, the women’s exact ages when they experienced infertility, pregnancy, miscarriage and stillbirth were not collected, so it was not feasible to know whether antidepressant medication use continued during the preconception period, pregnancy or at other times. Furthermore, this study included predominantly White women, which limited the generalizability of its findings to women of other ethnic backgrounds. Even though the data indicated the association between pre-existing depression and fertility issues increased with age, the small number of adverse fertility events in the late 30s and early 40s limited the power and resulted in relatively wide 95%CIs. Finally, selection bias might have existed. Women with a history of depression were more likely to be excluded from the analysis because they were less likely to complete the survey, to try to become pregnant, or to be pregnant, especially in the later surveys (Table S7–S9). In addition, 11.8%, 8.7% and 8.6% records were excluded owing to missing data in the analysis of infertility, miscarriage and stillbirth respectively, and women who were excluded from the analyses were less educated and more likely to be current smokers compared women included in the analyses.

## Conclusions

Women with a history of depression were at higher risk of subsequent infertility, miscarriage and recurrent miscarriages. The association between diagnosed depression and infertility may increase with age, especially in women’s late 30s and early 40s, and the association for miscarriage tended to be U-shaped, with stronger association in women’s early 20s, late 30s and early 40s. The findings suggest that health professionals should be mindful of these potentially increased risks of fertility issues when supporting women with a history of depression who are, or are planning to become pregnant. Future cohort studies with larger sample size and detailed data on antidepressant medication and dates of adverse pregnancy outcomes are needed to confirm current findings and reveal more information on the effects of antidepressant medications.

## Supporting information

Liang et al. supplementary material 1 Liang et al. supplementary material

Liang et al. supplementary material 2 Liang et al. supplementary material

## Data Availability

The ALSWH data are available on request to the ALSWH Data Access Committee. The guidelines on how to apply for access the data are at https://alswh.org.au/for-data-users/applying-for-data/.

## Supplementary material

The supplementary material for this article can be found at https://doi.org/10.1017/S2045796024000374.
