# Supplementary material for: Association of a history of depression with infertility, miscarriage and stillbirth: a longitudinal cohort study
Source: Epidemiol Psychiatr Sci. 2024 Nov 5;33:e55. doi: 10.1017/S2045796024000374 (PMC11561523; doi:10.1017/S2045796024000374)
Supplement: Liang et al. supplementary material 2 — Liang et al. supplementary material [file S2045796024000374sup002.docx]

**Association of a history of depression with infertility, miscarriage, and stillbirth: a longitudinal cohort study**

**Short title: depression, and infertility, miscarriage, and stillbirth**

Chen Liang^1^ (0000-0002-0023-1066), Hsin-Fang Chung^1^ (0000-0003-3261-5942), Annette J. Dobson^1^(0000-0003-4956-0124), and Gita D. Mishra^1^ (0000-0001-9610-5904)

^1^ The University of Queensland, School of Public Health

Contents

**[Figure S1.](#_Toc165451100)** [Flow diagram of the study population for infertility by survey 4](#_Toc165451100)

**[Figure S2.](#_Toc165451101)** [Flow diagram of the study population for miscarriage by survey 5](#_Toc165451101)

**[Figure S3.](#_Toc165451102)** [Flow diagram of the study population for stillbirth by survey 6](#_Toc165451102)

**[Figure S4.](#_Toc165451103)** [The association of history of depression diagnosis with and without anti-depressant medication with the risk of subsequent infertility, miscarriage, and recurrent miscarriages by survey 7](#_Toc165451103)

**[Table S1.](#_Toc165451104)** [Characteristics of women who were included in or excluded from the analysis of infertility due to missing data 9](#_Toc165451104)

**[Table S2.](#_Toc165451105)** [Characteristics of women who were included in or excluded from the analysis of miscarriage due to missing data 11](#_Toc165451105)

**[Table S3.](#_Toc165451106)** [Characteristics of women who were included in or excluded from the analysis of stillbirth due to missing data 14](#_Toc165451106)

**[Table S4.](#_Toc165451107)** [Sensitivity analysis of the association of history of depression (including medication), history of depression with and without anti-depressant medication with the risk of subsequent infertility, miscarriage, and stillbirth with additional adjustment of anxiety 16](#_Toc165451107)

**[Table S5.](#_Toc165451108)** [Sensitivity analysis of the association of history of depression (including medication), history of depression with and without anti-depressant medication with the risk of subsequent infertility, miscarriage, and stillbirth restricting to records free of any fertility issue at the current survey 17](#_Toc165451108)

**[Table S6.](#_Toc165451109)** [Sensitivity analysis of the association of history of depression (including medication), history of depression with and without anti-depressant medication with the risk of subsequent infertility, miscarriage, and stillbirth restricting to records with children in the current survey 18](#_Toc165451109)

**[Table S7.](#_Toc165451110)** [Dropout rate in the next survey among women with and without depression 19](#_Toc165451110)

**[Table S8.](#_Toc165451111)** [Proportion of women ever or never trying to be pregnant in the next survey among women with and without depression 19](#_Toc165451111)

**[Table S9.](#_Toc165451112)** [Proportion of women ever or never being pregnant in the next survey among women with and without depression 19](#_Toc165451112)

# **Figure S1.** Flow diagram of the study population for infertility by survey


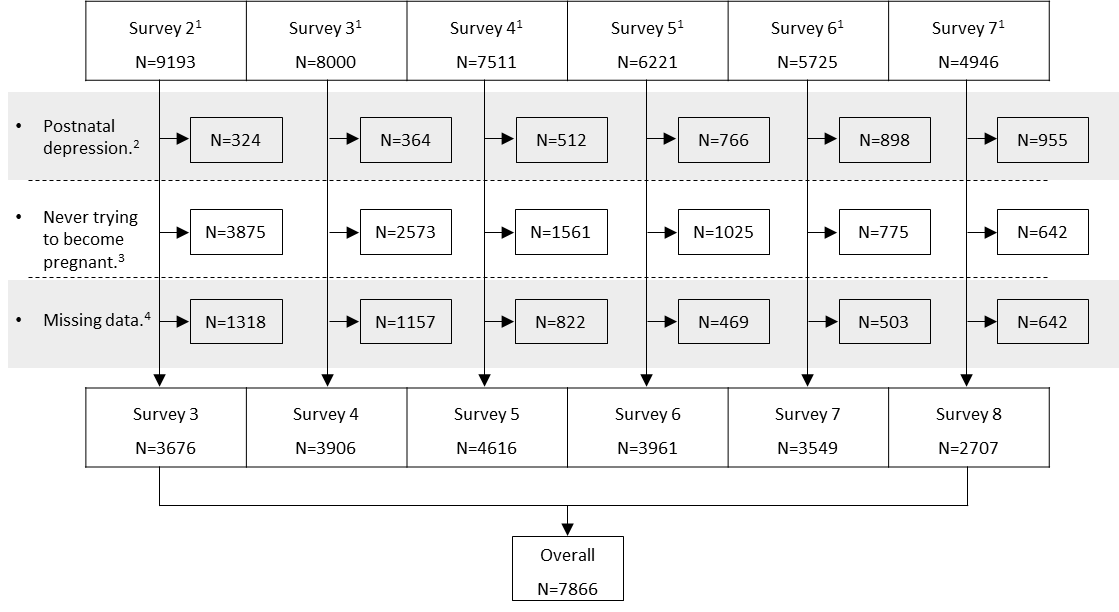


^1^: Women did not report history of infertility up to the current survey. ^2^: Women reported postnatal depression up to the current survey. ^3^: Women reported never trying to become pregnant in the next survey. ^4^: Women with missing data on depression, covariates, or one-survey lagged infertility.

# **Figure S2.** Flow diagram of the study population for miscarriage by survey


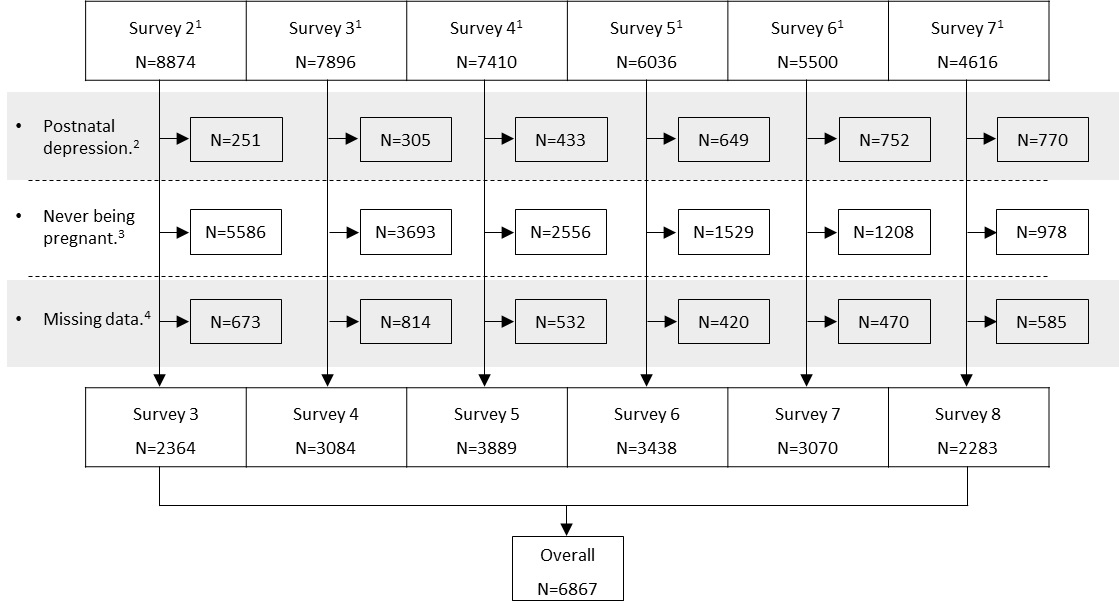


^1^: Women did not report history of miscarriage up to the current survey. ^2^: Women reported postnatal depression up to the current survey. ^3^: Women reported never being pregnant in the next survey. ^4^: Women with missing data on depression, covariates, or one-survey lagged miscarriage.

# **Figure S3.** Flow diagram of the study population for stillbirth by survey


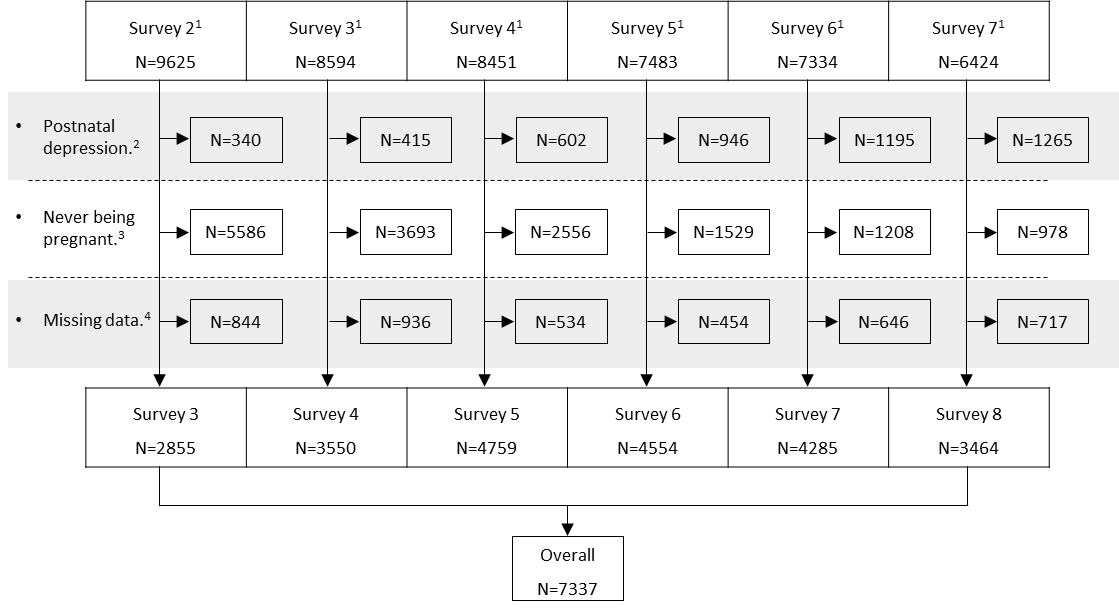


^1^: Women did not report history of history of stillbirth up to the current survey. ^2^: Women reported postnatal depression up to the current survey. ^3^: Women reported never being pregnant in the next survey. ^4^: Women with missing data on depression, covariates, or one-survey lagged stillbirth.

# **Figure S4.** The association of history of depression diagnosis with and without anti-depressant medication with the risk of subsequent infertility, miscarriage, and recurrent miscarriages by survey


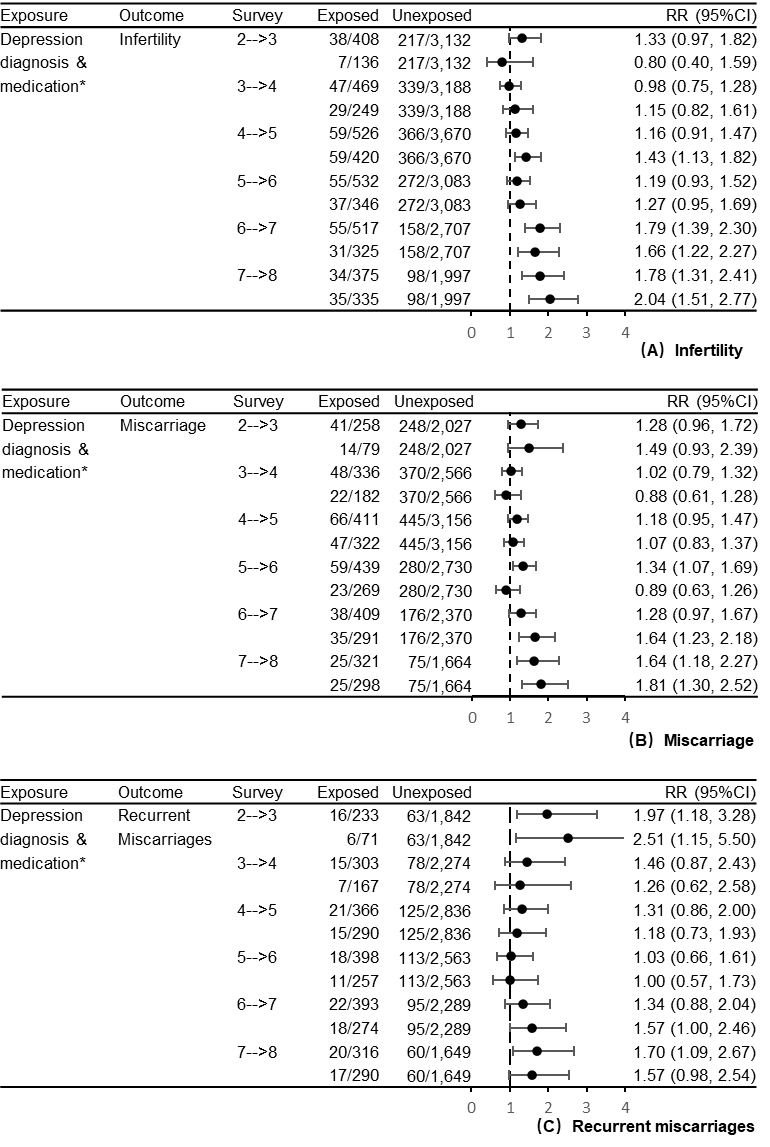


^*^ For each survey, the first row presents the results for women with a history of diagnosed depression and without anti-depressant medication vs non-depressed women, and the second row presents the results for women with a history of diagnosed depression and anti-depressant medication vs non-depressed women.

*Note.* Models were adjusted for education level (did not complete high school, completed high school, trade or certificate, and university or higher degree), marital status (married/defacto, divorced/separated/widowed, and single), body-mass index (<18.5, 18.5-22.9, 23.0-27.4, and ≥27.5 kg/m^2^ for Asian women; <18.5, 18.5-24.9, 25.0-29.9, and ≥30.0 kg/m^2^ for other women), smoking status (never smoker, past smoker, and current smoker), and alcohol intake [never drink, rarely drink, and drinker (i.e., ≤14, 15-28, and ≥29 drinks per week)]. The time trend and subject effect were taken into account by including a fixed effect of survey number and a clustering effect for subject. An interaction term between the exposure and survey number was included. In the analyses of infertility, p-value for the interaction term (depression & medication)*survey was 0.0132. In the analyses of miscarriage, p-value for the interaction term (depression & medication)*survey was 0.0553. In the analyses of recurrent miscarriages, p-value for the interaction term (depression & medication)*survey was 0.7147.

# **Table S1.** Characteristics of women who were included in or excluded from the analysis of infertility due to missing data

|  | Survey 2 |  | Survey3 |  | Survey 4 |  | Survey 5 |  | Survey 6 |  | Survey 7 |  |
| --- | --- | --- | --- | --- | --- | --- | --- | --- | --- | --- | --- | --- |
|  | Excluded | Included | Excluded | Included | Excluded | Included | Excluded | Included | Excluded | Included | Excluded | Included |
|  | N=1318 | N=3676 | N=1157 | N=3906 | N=822 | N=4616 | N=469 | N=3961 | N=503 | N=3549 | N=642 | N=2707 |
| Depression | |  |  |  |  |  |  |  |  |  |  |  |
| Never | 1140 (86.5%) | 3132 (85.2%) | 959 (82.9%) | 3188 (81.6%) | 653 (79.4%) | 3670 (79.5%) | 334 (71.2%) | 3083 (77.8%) | 340 (67.6%) | 2707 (76.3%) | 462 (72.0%) | 1997 (73.8%) |
| Ever | 178 (13.5%) | 544 (14.8%) | 198 (17.1%) | 718 (18.4%) | 169 (20.6%) | 946 (20.5%) | 135 (28.8%) | 878 (22.2%) | 163 (32.4%) | 842 (23.7%) | 180 (28.0%) | 710 (26.2%) |
| Anti-depressants | |  |  |  |  |  |  |  |  |  |  |  |
| Never | 1279 (97.0%) | 3540 (96.3%) | 1078 (93.2%) | 3657 (93.6%) | 744 (90.5%) | 4196 (90.9%) | 406 (86.6%) | 3615 (91.3%) | 416 (82.7%) | 3224 (90.8%) | 538 (83.8%) | 2372 (87.6%) |
| Ever | 39 (3.0%) | 136 (3.7%) | 79 (6.8%) | 249 (6.4%) | 78 (9.5%) | 420 (9.1%) | 63 (13.4%) | 346 (8.7%) | 87 (17.3%) | 325 (9.2%) | 104 (16.2%) | 335 (12.4%) |
| Education level | |  |  |  |  |  |  |  |  |  |  |  |
| Did not complete high school | 230 (17.5%) | 426 (11.6%) | 125 (10.8%) | 331 (8.5%) | 76 (9.2%) | 262 (5.7%) | 45 (9.6%) | 174 (4.4%) | 38 (7.6%) | 120 (3.4%) | 35 (5.5%) | 74 (2.7%) |
| Completed high school | 363 (27.5%) | 937 (25.5%) | 222 (19.2%) | 697 (17.8%) | 138 (16.8%) | 596 (12.9%) | 60 (12.8%) | 398 (10.0%) | 66 (13.1%) | 294 (8.3%) | 59 (9.2%) | 173 (6.4%) |
| Trade or diploma | 398 (30.2%) | 1042 (28.3%) | 402 (34.7%) | 1158 (29.6%) | 287 (34.9%) | 1373 (29.7%) | 176 (37.5%) | 1154 (29.1%) | 183 (36.4%) | 1009 (28.4%) | 213 (33.2%) | 763 (28.2%) |
| University or higher degree | 327 (24.8%) | 1271 (34.6%) | 408 (35.3%) | 1720 (44.0%) | 321 (39.1%) | 2385 (51.7%) | 188 (40.1%) | 2235 (56.4%) | 216 (42.9%) | 2126 (59.9%) | 335 (52.2%) | 1697 (62.7%) |
| Marital status | | | | | | | | | | | | |
| Married/defacto | 743 (57.5%) | 2074 (56.4%) | 821 (72.4%) | 2824 (72.3%) | 520 (65.0%) | 3718 (80.5%) | 318 (69.9%) | 3317 (83.7%) | 317 (67.9%) | 2999 (84.5%) | 393 (75.0%) | 2308 (85.3%) |
| Divorced/separated/widowed | 23 (1.8%) | 62 (1.7%) | 33 (2.9%) | 138 (3.5%) | 41 (5.1%) | 186 (4.0%) | 24 (5.3%) | 187 (4.7%) | 38 (8.1%) | 202 (5.7%) | 43 (8.2%) | 199 (7.4%) |
| Single | 527 (40.8%) | 1540 (41.9%) | 280 (24.7%) | 944 (24.2%) | 239 (29.9%) | 712 (15.4%) | 113 (24.8%) | 457 (11.5%) | 112 (24.0%) | 348 (9.8%) | 88 (16.8%) | 200 (7.4%) |
| Body-mass index^1^ | |  |  |  |  |  |  |  |  |  |  |  |
| <18.5 | 54 (7.9%) | 237 (6.4%) | 25 (4.9%) | 164 (4.2%) | 30 (4.2%) | 155 (3.4%) | 9 (2.2%) | 109 (2.8%) | 10 (2.2%) | 82 (2.3%) | 9 (1.8%) | 51 (1.9%) |
| 18.5-24.9 | 419 (61.3%) | 2207 (60.0%) | 282 (55.3%) | 2253 (57.7%) | 387 (54.6%) | 2547 (55.2%) | 207 (51.0%) | 2129 (53.7%) | 213 (46.7%) | 1825 (51.4%) | 233 (45.3%) | 1305 (48.2%) |
| 25.0-29.9 | 140 (20.5%) | 801 (21.8%) | 124 (24.3%) | 921 (23.6%) | 153 (21.6%) | 1202 (26.0%) | 95 (23.4%) | 1055 (26.6%) | 124 (27.2%) | 947 (26.7%) | 154 (30.0%) | 739 (27.3%) |
| ≥30.0 | 70 (10.2%) | 431 (11.7%) | 79 (15.5%) | 568 (14.5%) | 139 (19.6%) | 712 (15.4%) | 95 (23.4%) | 668 (16.9%) | 109 (23.9%) | 695 (19.6%) | 118 (23.0%) | 612 (22.6%) |
| Smoking status | |  |  |  |  |  |  |  |  |  |  |  |
| Never smoker | 644 (50.4%) | 1938 (52.7%) | 633 (55.6%) | 2194 (56.2%) | 422 (53.1%) | 2723 (59.0%) | 254 (54.7%) | 2397 (60.5%) | 287 (58.6%) | 2247 (63.3%) | 342 (59.7%) | 1748 (64.6%) |
| Past smoker | 246 (19.2%) | 598 (16.3%) | 265 (23.3%) | 757 (19.4%) | 173 (21.8%) | 1092 (23.7%) | 103 (22.2%) | 1056 (26.7%) | 123 (25.1%) | 975 (27.5%) | 158 (27.6%) | 727 (26.9%) |
| Current smoker | 388 (30.4%) | 1140 (31.0%) | 241 (21.2%) | 955 (24.4%) | 199 (25.1%) | 801 (17.4%) | 107 (23.1%) | 508 (12.8%) | 80 (16.3%) | 327 (9.2%) | 73 (12.7%) | 232 (8.6%) |
| Alcohol intake by NHMRC (drinks per week) | | | | |  |  |  |  |  |  |  |  |
| Non-drinker | 182 (14.2%) | 324 (8.8%) | 129 (11.3%) | 257 (6.6%) | 110 (13.9%) | 432 (9.4%) | 57 (12.6%) | 420 (10.6%) | 52 (10.8%) | 363 (10.2%) | 65 (11.3%) | 255 (9.4%) |
| Rarely drink | 448 (34.8%) | 1133 (30.8%) | 401 (35.2%) | 1029 (26.3%) | 209 (26.3%) | 1155 (25.0%) | 129 (28.6%) | 908 (22.9%) | 128 (26.5%) | 853 (24.0%) | 141 (24.5%) | 588 (21.7%) |
| ≤14, 15-28, or ≥29 | 656 (51.0%) | 2219 (60.4%) | 609 (53.5%) | 2620 (67.1%) | 475 (59.8%) | 3029 (65.6%) | 265 (58.8%) | 2633 (66.5%) | 303 (62.7%) | 2333 (65.7%) | 370 (64.2%) | 1864 (68.9%) |
| Infertility^2^ |  |  |  |  |  |  |  |  |  |  |  |  |
| Never | 547 (95.3%) | 3414 (92.9%) | 576 (95.7%) | 3491 (89.4%) | 118 (88.1%) | 4132 (89.5%) | 69 (90.8%) | 3597 (90.8%) | 55 (98.2%) | 3305 (93.1%) | 161 (95.3%) | 2540 (93.8%) |
| Ever | 27 (4.7%) | 262 (7.1%) | 26 (4.3%) | 415 (10.6%) | 16 (11.9%) | 484 (10.5%) | 7 (9.2%) | 364 (9.2%) | 1 (1.8%) | 244 (6.9%) | 8 (4.7%) | 167 (6.2%) |

^1^ For Asian women, body-mass index was categorized as <18.5, 18.5-22.9, 23.0-27.4, and ≥27.5 kg/m^2^. ^2^ Infertility in the next survey.

# **Table S2.** Characteristics of women who were included in or excluded from the analysis of miscarriage due to missing data

|  | Survey 2 |  | Survey3 |  | Survey 4 |  | Survey 5 |  | Survey 6 |  | Survey 7 |  |
| --- | --- | --- | --- | --- | --- | --- | --- | --- | --- | --- | --- | --- |
|  | Excluded | Included | Excluded | Included | Excluded | Included | Excluded | Included | Excluded | Included | Excluded | Included |
|  | N=673 | N=2364 | N=814 | N=3084 | N=532 | N=3889 | N=420 | N=3438 | N=469 | N=3070 | N=585 | N=2283 |
| Depression | |  |  |  |  |  |  |  |  |  |  |  |
| Never | 591 (87.8%) | 2027 (85.7%) | 694 (85.3%) | 2566 (83.2%) | 429 (80.6%) | 3156 (81.2%) | 336 (80.0%) | 2730 (79.4%) | 358 (76.3%) | 2370 (77.2%) | 449 (76.8%) | 1664 (72.9%) |
| Ever | 82 (12.2%) | 337 (14.3%) | 120 (14.7%) | 518 (16.8%) | 103 (19.4%) | 733 (18.8%) | 84 (20.0%) | 708 (20.6%) | 111 (23.7%) | 700 (22.8%) | 136 (23.2%) | 619 (27.1%) |
| Anti-depressant | |  |  |  |  |  |  |  |  |  |  |  |
| Never | 660 (98.1%) | 2285 (96.7%) | 769 (94.5%) | 2902 (94.1%) | 482 (90.6%) | 3567 (91.7%) | 382 (91.0%) | 3169 (92.2%) | 413 (88.1%) | 2779 (90.5%) | 513 (87.7%) | 1985 (86.9%) |
| Ever | 13 (1.9%) | 79 (3.3%) | 45 (5.5%) | 182 (5.9%) | 50 (9.4%) | 322 (8.3%) | 38 (9.0%) | 269 (7.8%) | 56 (11.9%) | 291 (9.5%) | 72 (12.3%) | 298 (13.1%) |
| Education level | |  |  |  |  |  |  |  |  |  |  |  |
| Did not complete high school | 151 (22.4%) | 317 (13.4%) | 105 (12.9%) | 285 (9.2%) | 80 (15.0%) | 224 (5.8%) | 43 (10.2%) | 147 (4.3%) | 38 (8.1%) | 107 (3.5%) | 34 (5.8%) | 61 (2.7%) |
| Completed high school | 191 (28.4%) | 654 (27.7%) | 170 (20.9%) | 588 (19.1%) | 108 (20.3%) | 535 (13.8%) | 70 (16.7%) | 362 (10.5%) | 65 (13.9%) | 262 (8.5%) | 46 (7.9%) | 157 (6.9%) |
| Trade or diploma | 222 (33.0%) | 702 (29.7%) | 287 (35.3%) | 967 (31.4%) | 209 (39.3%) | 1210 (31.1%) | 174 (41.4%) | 999 (29.1%) | 177 (37.7%) | 869 (28.3%) | 182 (31.1%) | 636 (27.9%) |
| University or higher degree | 109 (16.2%) | 691 (29.2%) | 252 (31.0%) | 1244 (40.3%) | 135 (25.4%) | 1920 (49.4%) | 133 (31.7%) | 1930 (56.1%) | 189 (40.3%) | 1832 (59.7%) | 323 (55.2%) | 1429 (62.6%) |
| Marital status | | | | | | | | | | | | |
| Married/defacto | 501 (76.5%) | 1506 (63.7%) | 677 (85.2%) | 2361 (76.6%) | 415 (80.7%) | 3300 (84.9%) | 354 (86.1%) | 2995 (87.1%) | 357 (81.9%) | 2651 (86.4%) | 422 (84.6%) | 1964 (86.0%) |
| Divorced/separated/widowed | 14 (2.1%) | 44 (1.9%) | 32 (4.0%) | 121 (3.9%) | 35 (6.8%) | 159 (4.1%) | 20 (4.9%) | 156 (4.5%) | 40 (9.2%) | 188 (6.1%) | 48 (9.6%) | 168 (7.4%) |
| Single | 140 (21.4%) | 814 (34.4%) | 86 (10.8%) | 602 (19.5%) | 64 (12.5%) | 430 (11.1%) | 37 (9.0%) | 287 (8.3%) | 39 (8.9%) | 231 (7.5%) | 29 (5.8%) | 151 (6.6%) |
| Body-mass index^1^ | |  |  |  |  |  |  |  |  |  |  |  |
| <18.5 | 14 (6.1%) | 152 (6.4%) | 10 (4.3%) | 123 (4.0%) | 15 (3.5%) | 129 (3.3%) | 10 (2.8%) | 80 (2.3%) | 8 (1.9%) | 67 (2.2%) | 11 (2.3%) | 39 (1.7%) |
| 18.5-24.9 | 134 (58.5%) | 1378 (58.3%) | 129 (55.8%) | 1740 (56.4%) | 221 (52.1%) | 2167 (55.7%) | 159 (44.8%) | 1867 (54.3%) | 185 (43.8%) | 1602 (52.2%) | 235 (49.7%) | 1124 (49.2%) |
| 25.0-29.9 | 59 (25.8%) | 553 (23.4%) | 55 (23.8%) | 759 (24.6%) | 94 (22.2%) | 993 (25.5%) | 91 (25.6%) | 916 (26.6%) | 131 (31.0%) | 791 (25.8%) | 127 (26.8%) | 612 (26.8%) |
| ≥30.0 | 22 (9.6%) | 281 (11.9%) | 37 (16.0%) | 462 (15.0%) | 94 (22.2%) | 600 (15.4%) | 95 (26.8%) | 575 (16.7%) | 98 (23.2%) | 610 (19.9%) | 100 (21.1%) | 508 (22.3%) |
| Smoking status | |  |  |  |  |  |  |  |  |  |  |  |
| Never smoker | 293 (45.2%) | 1216 (51.4%) | 451 (56.3%) | 1709 (55.4%) | 234 (45.8%) | 2318 (59.6%) | 208 (49.9%) | 2131 (62.0%) | 258 (56.6%) | 1967 (64.1%) | 321 (58.8%) | 1475 (64.6%) |
| Past smoker | 155 (23.9%) | 404 (17.1%) | 197 (24.6%) | 606 (19.6%) | 127 (24.9%) | 937 (24.1%) | 113 (27.1%) | 913 (26.6%) | 122 (26.8%) | 829 (27.0%) | 171 (31.3%) | 616 (27.0%) |
| Current smoker | 200 (30.9%) | 744 (31.5%) | 153 (19.1%) | 769 (24.9%) | 150 (29.4%) | 634 (16.3%) | 96 (23.0%) | 394 (11.5%) | 76 (16.7%) | 274 (8.9%) | 54 (9.9%) | 192 (8.4%) |
| Alcohol intake by NHMRC (drinks per week) | | | | |  |  |  |  |  |  |  |  |
| Non-drinker | 114 (17.5%) | 222 (9.4%) | 102 (12.8%) | 229 (7.4%) | 80 (15.7%) | 382 (9.8%) | 57 (14.2%) | 392 (11.4%) | 53 (11.7%) | 305 (9.9%) | 57 (10.5%) | 197 (8.6%) |
| Rarely drink | 261 (40.2%) | 754 (31.9%) | 292 (36.5%) | 845 (27.4%) | 152 (29.8%) | 1012 (26.0%) | 106 (26.4%) | 804 (23.4%) | 139 (30.7%) | 744 (24.2%) | 137 (25.1%) | 499 (21.9%) |
| ≤14, 15-28, or ≥29 | 275 (42.3%) | 1388 (58.7%) | 406 (50.8%) | 2010 (65.2%) | 278 (54.5%) | 2495 (64.2%) | 239 (59.5%) | 2242 (65.2%) | 261 (57.6%) | 2021 (65.8%) | 351 (64.4%) | 1587 (69.5%) |
| Miscarriage^2^ | |  |  |  |  |  |  |  |  |  |  |  |
| Never | 391 (89.3%) | 2061 (87.2%) | 492 (88.3%) | 2644 (85.7%) | 95 (86.4%) | 3331 (85.7%) | 61 (91.0%) | 3076 (89.5%) | 44 (95.7%) | 2821 (91.9%) | 129 (92.8%) | 2158 (94.5%) |
| Ever | 47 (10.7%) | 303 (12.8%) | 65 (11.7%) | 440 (14.3%) | 15 (13.6%) | 558 (14.3%) | 6 (9.0%) | 362 (10.5%) | 2 (4.3%) | 249 (8.1%) | 10 (7.2%) | 125 (5.5%) |
| No. of miscarriage^2^ | |  |  |  |  |  |  |  |  |  |  |  |
| 0 | 391 (89.9%) | 2061 (87.7%) | 492 (89.3%) | 2644 (86.9%) | 95 (88.0%) | 3331 (86.9%) | 61 (91.0%) | 3076 (89.5%) | 44 (97.8%) | 2821 (91.9%) | 129 (92.8%) | 2158 (94.5%) |
| 1 | 37 (8.5%) | 246 (10.5%) | 50 (9.1%) | 348 (11.4%) | 12 (11.1%) | 417 (10.9%) | 6 (9.0%) | 299 (8.7%) | 1 (2.2%) | 193 (6.3%) | 8 (5.8%) | 82 (3.6%) |
| ≥2 | 7 (1.6%) | 43 (1.8%) | 9 (1.6%) | 52 (1.7%) | 1 (0.9%) | 83 (2.2%) | 0 (0.0%) | 63 (1.8%) | 0 (0.0%) | 56 (1.8%) | 2 (1.4%) | 43 (1.9%) |

^1^ For Asian women, body-mass index was categorized as <18.5, 18.5-22.9, 23.0-27.4, and ≥27.5 kg/m^2^. ^2^ Miscarriage or No. of miscarriage in the next survey.

# **Table S3.** Characteristics of women who were included in or excluded from the analysis of stillbirth due to missing data

|  | Survey 2 |  | Survey3 |  | Survey 4 |  | Survey 5 |  | Survey 6 |  | Survey 7 |  |
| --- | --- | --- | --- | --- | --- | --- | --- | --- | --- | --- | --- | --- |
|  | Excluded | Included | Excluded | Included | Excluded | Included | Excluded | Included | Excluded | Included | Excluded | Included |
|  | N=844 | N=2855 | N=936 | N=3550 | N=534 | N=4759 | N=454 | N=4554 | N=646 | N=4285 | N=717 | N=3464 |
| Depression | |  |  |  |  |  |  |  |  |  |  |  |
| Never | 739 (87.6%) | 2408 (84.3%) | 792 (84.6%) | 2879 (81.1%) | 430 (80.5%) | 3761 (79.0%) | 349 (76.9%) | 3542 (77.8%) | 480 (74.3%) | 3254 (75.9%) | 529 (73.8%) | 2514 (72.6%) |
| Ever | 105 (12.4%) | 447 (15.7%) | 144 (15.4%) | 671 (18.9%) | 104 (19.5%) | 998 (21.0%) | 105 (23.1%) | 1012 (22.2%) | 166 (25.7%) | 1031 (24.1%) | 188 (26.2%) | 950 (27.4%) |
| Anti-depressant | |  |  |  |  |  |  |  |  |  |  |  |
| Never | 827 (98.0%) | 2746 (96.2%) | 890 (95.1%) | 3323 (93.6%) | 491 (91.9%) | 4323 (90.8%) | 409 (90.1%) | 4162 (91.4%) | 564 (87.3%) | 3872 (90.4%) | 619 (86.3%) | 3002 (86.7%) |
| Ever | 17 (2.0%) | 109 (3.8%) | 46 (4.9%) | 227 (6.4%) | 43 (8.1%) | 436 (9.2%) | 45 (9.9%) | 392 (8.6%) | 82 (12.7%) | 413 (9.6%) | 98 (13.7%) | 462 (13.3%) |
| Education level | |  |  |  |  |  |  |  |  |  |  |  |
| Did not complete high school | 208 (24.6%) | 421 (14.7%) | 117 (12.5%) | 359 (10.1%) | 79 (14.8%) | 306 (6.4%) | 41 (9.0%) | 222 (4.9%) | 45 (7.0%) | 155 (3.6%) | 38 (5.3%) | 98 (2.8%) |
| Completed high school | 234 (27.7%) | 794 (27.8%) | 206 (22.0%) | 682 (19.2%) | 99 (18.5%) | 684 (14.4%) | 76 (16.7%) | 493 (10.8%) | 82 (12.7%) | 376 (8.8%) | 67 (9.3%) | 230 (6.6%) |
| Trade or diploma | 281 (33.3%) | 846 (29.6%) | 328 (35.0%) | 1130 (31.8%) | 210 (39.3%) | 1513 (31.8%) | 188 (41.4%) | 1357 (29.8%) | 236 (36.5%) | 1242 (29.0%) | 233 (32.5%) | 978 (28.2%) |
| University or higher degree | 121 (14.3%) | 794 (27.8%) | 285 (30.4%) | 1379 (38.8%) | 146 (27.3%) | 2256 (47.4%) | 149 (32.8%) | 2482 (54.5%) | 283 (43.8%) | 2512 (58.6%) | 379 (52.9%) | 2158 (62.3%) |
| Marital status | | | | | | | | | | | | |
| Married/defacto | 629 (76.3%) | 1816 (63.6%) | 799 (87.5%) | 2708 (76.3%) | 423 (82.6%) | 4053 (85.2%) | 387 (88.2%) | 3982 (87.4%) | 522 (85.4%) | 3724 (86.9%) | 494 (84.0%) | 3013 (87.0%) |
| Divorced/separated/widowed | 19 (2.3%) | 64 (2.2%) | 34 (3.7%) | 145 (4.1%) | 31 (6.1%) | 203 (4.3%) | 17 (3.9%) | 219 (4.8%) | 47 (7.7%) | 276 (6.4%) | 60 (10.2%) | 248 (7.2%) |
| Single | 176 (21.4%) | 975 (34.2%) | 80 (8.8%) | 697 (19.6%) | 58 (11.3%) | 503 (10.6%) | 35 (8.0%) | 353 (7.8%) | 42 (6.9%) | 285 (6.7%) | 34 (5.8%) | 203 (5.9%) |
| Body-mass index^1^ | |  |  |  |  |  |  |  |  |  |  |  |
| <18.5 | 18 (6.6%) | 191 (6.7%) | 9 (3.6%) | 145 (4.1%) | 12 (2.8%) | 164 (3.4%) | 11 (2.8%) | 121 (2.7%) | 14 (2.4%) | 101 (2.4%) | 9 (1.6%) | 67 (1.9%) |
| 18.5-24.9 | 160 (59.0%) | 1626 (57.0%) | 135 (53.8%) | 1971 (55.5%) | 219 (51.5%) | 2578 (54.2%) | 193 (49.1%) | 2408 (52.9%) | 287 (48.9%) | 2187 (51.0%) | 262 (46.3%) | 1678 (48.4%) |
| 25.0-29.9 | 67 (24.7%) | 674 (23.6%) | 67 (26.7%) | 862 (24.3%) | 108 (25.4%) | 1230 (25.8%) | 97 (24.7%) | 1220 (26.8%) | 168 (28.6%) | 1126 (26.3%) | 156 (27.6%) | 928 (26.8%) |
| ≥30.0 | 26 (9.6%) | 364 (12.7%) | 40 (15.9%) | 572 (16.1%) | 86 (20.2%) | 787 (16.5%) | 92 (23.4%) | 805 (17.7%) | 118 (20.1%) | 871 (20.3%) | 139 (24.6%) | 791 (22.8%) |
| Smoking status | |  |  |  |  |  |  |  |  |  |  |  |
| Never smoker | 371 (45.6%) | 1377 (48.2%) | 504 (54.8%) | 1916 (54.0%) | 246 (48.1%) | 2781 (58.4%) | 236 (52.7%) | 2786 (61.2%) | 353 (56.2%) | 2704 (63.1%) | 392 (60.1%) | 2221 (64.1%) |
| Past smoker | 189 (23.2%) | 506 (17.7%) | 243 (26.4%) | 716 (20.2%) | 118 (23.1%) | 1163 (24.4%) | 113 (25.2%) | 1216 (26.7%) | 172 (27.4%) | 1194 (27.9%) | 192 (29.4%) | 956 (27.6%) |
| Current smoker | 253 (31.1%) | 972 (34.0%) | 173 (18.8%) | 918 (25.9%) | 147 (28.8%) | 815 (17.1%) | 99 (22.1%) | 552 (12.1%) | 103 (16.4%) | 387 (9.0%) | 68 (10.4%) | 287 (8.3%) |
| Alcohol intake by NHMRC (drinks per week) | | | | |  |  |  |  |  |  |  |  |
| Non-drinker | 153 (18.7%) | 279 (9.8%) | 123 (13.4%) | 269 (7.6%) | 76 (14.9%) | 482 (10.1%) | 60 (13.9%) | 540 (11.9%) | 86 (13.8%) | 460 (10.7%) | 75 (11.5%) | 322 (9.3%) |
| Rarely drink | 333 (40.7%) | 936 (32.8%) | 350 (38.1%) | 1015 (28.6%) | 155 (30.5%) | 1290 (27.1%) | 136 (31.4%) | 1095 (24.0%) | 161 (25.8%) | 1077 (25.1%) | 179 (27.4%) | 780 (22.5%) |
| ≤14, 15-28, or ≥29 | 332 (40.6%) | 1640 (57.4%) | 445 (48.5%) | 2266 (63.8%) | 278 (54.6%) | 2987 (62.8%) | 237 (54.7%) | 2919 (64.1%) | 378 (60.5%) | 2748 (64.1%) | 400 (61.2%) | 2362 (68.2%) |
| Stillbirth^2^ |  |  |  |  |  |  |  |  |  |  |  |  |
| Never | 548 (97.9%) | 2820 (98.8%) | 643 (98.0%) | 3490 (98.3%) | 131 (98.5%) | 4660 (97.9%) | 81 (100.0%) | 4538 (99.6%) | 63 (100.0%) | 4257 (99.3%) | 217 (100.0%) | 3455 (99.7%) |
| Ever | 12 (2.1%) | 35 (1.2%) | 13 (2.0%) | 60 (1.7%) | 2 (1.5%) | 99 (2.1%) | 0 (0.0%) | 16 (0.4%) | 0 (0.0%) | 28 (0.7%) | 0 (0.0%) | 9 (0.3%) |

# ^1^ For Asian women, body-mass index was categorized as <18.5, 18.5-22.9, 23.0-27.4, and ≥27.5 kg/m^2^. ^2^ Stillbirth in the next survey.**Table S4.** Sensitivity analysis of the association of history of depression (including medication), history of depression with and without anti-depressant medication with the risk of subsequent infertility, miscarriage, and stillbirth with additional adjustment of anxiety


| Exposure |  | Crude model | Adjusted model 1 | Adjusted model 2 | Adjusted model 3 |
| --- | --- | --- | --- | --- | --- |
| Outcome=infertility | |  |  |  |  |
| Depression | Never | Ref. | Ref. | Ref. | Ref. |
|  | Ever | 1.30 (1.18, 1.43) | 1.35 (1.22, 1.50) | 1.35 (1.22, 1.50) | 1.30 (1.17, 1.46) |
| Depression | Never & never | Ref. | Ref. | Ref. | Ref. |
| diagnosis & | Ever & never | 1.26 (1.12, 1.42) | 1.31 (1.16, 1.48) | 1.31 (1.16, 1.47) | 1.27 (1.12, 1.44) |
| medication | Ever & ever | 1.36 (1.18, 1.56) | 1.45 (1.25, 1.67) | 1.43 (1.24, 1.65) | 1.37 (1.17, 1.60) |
| Outcome=miscarriage | |  |  |  |  |
| Depression | Never | Ref. | Ref. | Ref. | Ref. |
|  | Ever | 1.16 (1.06, 1.29) | 1.21 (1.09, 1.34) | 1.22 (1.10, 1.34) | 1.21 (1.09, 1.35) |
| Depression | Never & never | Ref. | Ref. | Ref. | Ref. |
| diagnosis & | Ever & never | 1.18 (1.04, 1.32) | 1.21 (1.08, 1.37) | 1.22 (1.08, 1.38) | 1.22 (1.08, 1.38) |
| medication | Ever & ever | 1.15 (0.99, 1.33) | 1.20 (1.04, 1.39) | 1.21 (1.04, 1.40) | 1.21 (1.03, 1.42) |
| Outcome=recurrent miscarriage | | |  |  |  |
| Depression | Never | Ref. | Ref. | Ref. | Ref. |
|  | Ever | 1.37 (1.16, 1.62) | 1.45 (1.22, 1.71) | 1.39 (1.17, 1.65) | 1.33 (1.11, 1.59) |
| Depression | Never & never | Ref. | Ref. | Ref. | Ref. |
| diagnosis & | Ever & never | 1.39 (1.14, 1.70) | 1.45 (1.18, 1.77) | 1.40 (1.14, 1.72) | 1.35 (1.10, 1.67) |
| medication | Ever & ever | 1.35 (1.06, 1.72) | 1.45 (1.13, 1.84) | 1.37 (1.07, 1.75) | 1.28 (0.99, 1.66) |
| Outcome=stillbirth | |  |  |  |  |
| Depression | Never | Ref. | Ref. | Ref. | Ref. |
|  | Ever | 1.04 (0.76, 1.42) | 0.97 (0.71, 1.33) | 0.96 (0.70, 1.32) | 0.90 (0.64, 1.27) |
| Depression | Never & never | Ref. | Ref. | Ref. | Ref. |
| diagnosis & | Ever & never | 0.91 (0.62, 1.36) | 0.86 (0.58, 1.28) | 0.85 (0.57, 1.27) | 0.82 (0.55, 1.22) |
| medication | Ever & ever | 1.25 (0.81, 1.94) | 1.15 (0.74, 1.80) | 1.14 (0.72, 1.79) | 1.05 (0.63, 1.75) |

Crude model took time trend and subject effect were taken into account by including a fixed effect of survey number and a clustering effect for subject. Adjusted model 1 was adjusted for education level (did not complete high school, completed high school, trade or certificate, and university or higher degree) and marital status (married/defacto, divorced/separated/widowed, and single). Adjusted model 2 was additionally adjusted for body-mass index (<18.5, 18.5-22.9, 23.0-27.4, and ≥27.5 kg/m^2^ for Asian women; <18.5, 18.5-24.9, 25.0-29.9, and ≥30.0 kg/m^2^ for other women), smoking status (never smoker, past smoker, and current smoker), and alcohol intake [non-drink, rarely drink, and other (i.e., ≤14, 15-28, and ≥29 drinks per week)]. Adjusted model 3 was additionally adjusted for anxiety (ever and never). No interaction term was included in any of the above models.

# **Table S5.** Sensitivity analysis of the association of history of depression (including medication), history of depression with and without anti-depressant medication with the risk of subsequent infertility, miscarriage, and stillbirth restricting to records free of any fertility issue at the current survey


| Exposure |  | Crude model | Adjusted model 1 | Adjusted model 2 |
| --- | --- | --- | --- | --- |
| Outcome=infertility | |  |  |  |
| Depression | Never | Ref. | Ref. | Ref. |
|  | Ever | 1.27 (1.14, 1.42) | 1.33 (1.18, 1.48) | 1.33 (1.18, 1.48) |
| Depression | Never & never | Ref. | Ref. | Ref. |
| diagnosis & | Ever & never | 1.18 (1.03, 1.36) | 1.23 (1.07, 1.41) | 1.23 (1.07, 1.41) |
| medication | Ever & ever | 1.41 (1.20, 1.65) | 1.50 (1.28, 1.75) | 1.49 (1.27, 1.75) |
| Outcome=miscarriage | |  |  |  |
| Depression | Never | Ref. | Ref. | Ref. |
|  | Ever | 1.21 (1.09, 1.35) | 1.25 (1.12, 1.40) | 1.27 (1.13, 1.42) |
| Depression | Never & never | Ref. | Ref. | Ref. |
| diagnosis & | Ever & never | 1.17 (1.02, 1.33) | 1.19 (1.04, 1.37) | 1.21 (1.05, 1.39) |
| medication | Ever & ever | 1.29 (1.10, 1.52) | 1.36 (1.15, 1.60) | 1.38 (1.17, 1.62) |
| Outcome=recurrent miscarriage | | |  |  |
| Depression | Never | Ref. | Ref. | Ref. |
|  | Ever | 1.51 (1.23, 1.86) | 1.63 (1.33, 2.01) | 1.58 (1.28, 1.94) |
| Depression | Never & never | Ref. | Ref. | Ref. |
| diagnosis & | Ever & never | 1.51 (1.18, 1.93) | 1.60 (1.25, 2.05) | 1.55 (1.21, 1.99) |
| medication | Ever & ever | 1.53 (1.13, 2.07) | 1.70 (1.25, 2.30) | 1.63 (1.20, 2.21) |
| Outcome=stillbirth | |  |  |  |
| Depression | Never | Ref. | Ref. | Ref. |
|  | Ever | 1.08 (0.73, 1.62) | 1.00 (0.67, 1.51) | 0.99 (0.65, 1.50) |
| Depression | Never & never | Ref. | Ref. | Ref. |
| diagnosis & | Ever & never | 1.01 (0.61, 1.65) | 0.94 (0.58, 1.54) | 0.92 (0.56, 1.53) |
| medication | Ever & ever | 1.23 (0.68, 2.23) | 1.12 (0.60, 2.07) | 1.10 (0.59, 2.06) |

Crude model took time trend and subject effect were taken into account by including a fixed effect of survey number and a clustering effect for subject. Adjusted model 1 was adjusted for education level (did not complete high school, completed high school, trade or certificate, and university or higher degree) and marital status (married/defacto, divorced/separated/widowed, and single). Adjusted model 2 was additionally adjusted for body-mass index (<18.5, 18.5-22.9, 23.0-27.4, and ≥27.5 kg/m^2^ for Asian women; <18.5, 18.5-24.9, 25.0-29.9, and ≥30.0 kg/m^2^ for other women), smoking status (never smoker, past smoker, and current smoker), and alcohol intake [non-drink, rarely drink, and other (i.e., ≤14, 15-28, and ≥29 drinks per week)]. No interaction term was included in any of the above models.

# **Table S6.** Sensitivity analysis of the association of history of depression (including medication), history of depression with and without anti-depressant medication with the risk of subsequent infertility, miscarriage, and stillbirth restricting to records with children in the current survey


| Exposure |  | Crude model | Adjusted model 1 | Adjusted model 2 |
| --- | --- | --- | --- | --- |
| Outcome=infertility | |  |  |  |
| Depression | Never | Ref. | Ref. | Ref. |
|  | Ever | 1.32 (1.08, 1.62) | 1.36 (1.10, 1.67) | 1.36 (1.10, 1.67) |
| Depression | Never & never | Ref. | Ref. | Ref. |
| diagnosis & | Ever & never | 1.48 (1.17, 1.87) | 1.52 (1.20, 1.92) | 1.51 (1.19, 1.91) |
| medication | Ever & ever | 1.06 (0.76, 1.49) | 1.12 (0.79, 1.57) | 1.10 (0.78, 1.54) |
| Outcome=miscarriage | |  |  |  |
| Depression | Never | Ref. | Ref. | Ref. |
|  | Ever | 1.09 (0.92, 1.28) | 1.14 (0.96, 1.35) | 1.14 (0.96, 1.35) |
| Depression | Never & never | Ref. | Ref. | Ref. |
| diagnosis & | Ever & never | 1.16 (0.95, 1.41) | 1.21 (0.99, 1.47) | 1.21 (0.99, 1.47) |
| medication | Ever & ever | 0.97 (0.74, 1.26) | 1.02 (0.78, 1.33) | 1.02 (0.78, 1.33) |
| Outcome=recurrent miscarriage | | |  |  |
| Depression | Never | Ref. | Ref. | Ref. |
|  | Ever | 1.16 (0.91, 1.47) | 1.21 (0.95, 1.54) | 1.17 (0.91, 1.49) |
| Depression | Never & never | Ref. | Ref. | Ref. |
| diagnosis & | Ever & never | 1.19 (0.89, 1.59) | 1.23 (0.92, 1.65) | 1.20 (0.89, 1.62) |
| medication | Ever & ever | 1.11 (0.77, 1.59) | 1.17 (0.82, 1.69) | 1.12 (0.78, 1.61) |
| Outcome=stillbirth | |  |  |  |
| Depression | Never | Ref. | Ref. | Ref. |
|  | Ever | 1.18 (0.73, 1.91) | 1.10 (0.67, 1.81) | 1.06 (0.65, 1.75) |
| Depression | Never & never | Ref. | Ref. | Ref. |
| diagnosis & | Ever & never | 1.24 (0.70, 2.18) | 1.17 (0.66, 2.07) | 1.13 (0.64, 1.99) |
| medication | Ever & ever | 1.07 (0.49, 2.33) | 0.99 (0.45, 2.18) | 0.96 (0.43, 2.11) |

Crude model took time trend and subject effect were taken into account by including a fixed effect of survey number and a clustering effect for subject. Adjusted model 1 was adjusted for education level (did not complete high school, completed high school, trade or certificate, and university or higher degree) and marital status (married/defacto, divorced/separated/widowed, and single). Adjusted model 2 was additionally adjusted for body-mass index (<18.5, 18.5-22.9, 23.0-27.4, and ≥27.5 kg/m^2^ for Asian women; <18.5, 18.5-24.9, 25.0-29.9, and ≥30.0 kg/m^2^ for other women), smoking status (never smoker, past smoker, and current smoker), and alcohol intake [non-drink, rarely drink, and other (i.e., ≤14, 15-28, and ≥29 drinks per week)]. No interaction term was included in any of the above models.

# **Table S7.** Dropout rate in the next survey among women with and without depression

| Survey | Without depression | |  | With depression | |
| --- | --- | --- | --- | --- | --- |
|  | Follow-up | Drop-out |  | Follow-up | Drop-out |
| 2🡪3 | 6,607 (80.5) | 1,598 (19.5) |  | 1,183 (79.8) | 300 (20.2) |
| 3🡪4 | 6,174 (85.9) | 1,014 (14.1) |  | 1,572 (83.0) | 321 (17.0) |
| 4🡪5 | 5,455 (80.1) | 1,355 (19.9) |  | 1,825 (78.2) | 510 (21.8) |
| 5🡪6 | 4,909 (86.1) | 792 (13.9) |  | 2,089 (83.6) | 409 (16.4) |
| 6🡪7 | 4,328 (82.7) | 904 (17.3) |  | 2,239 (80.6) | 538 (19.4) |
| 7🡪8 | 3,794 (85.4) | 648 (14.6) |  | 2,364 (86.2) | 380 (13.8) |

All data are presented as No. (%).

# **Table S8.** Proportion of women ever or never trying to be pregnant in the next survey among women with and without depression

| Survey | Without depression | |  | With depression | |
| --- | --- | --- | --- | --- | --- |
|  | Ever try to  be pregnant | Never try to  be pregnant |  | Ever try to  be pregnant | Never try to  be pregnant |
| 2🡪3 | 4,045 (54.6) | 3,368 (45.4) |  | 800 (60.0) | 533 (40.0) |
| 3🡪4 | 4,334 (68.6) | 1,985 (31.4) |  | 1,164 (70.0) | 499 (30.0) |
| 4🡪5 | 4,733 (81.1) | 1,104 (18.9) |  | 1,631 (79.9) | 410 (20.1) |
| 5🡪6 | 4,431 (87.0) | 663 (13.0) |  | 1,880 (84.7) | 339 (15.3) |
| 6🡪7 | 4,204 (89.8) | 476 (10.2) |  | 2,184 (88.3) | 288 (11.7) |
| 7🡪8 | 3,534 (90.2) | 383 (9.8) |  | 2,190 (89.2) | 266 (10.8) |

All data are presented as No. (%).

# **Table S9.** Proportion of women ever or never being pregnant in the next survey among women with and without depression

| Survey | Without depression | |  | With depression | |
| --- | --- | --- | --- | --- | --- |
|  | Ever been  pregnant | Never been  pregnant |  | Ever been  pregnant | Never been  pregnant |
| 2🡪3 | 3,051 (75.4) | 993 (24.6) |  | 640 (80.0) | 160 (20.0) |
| 3🡪4 | 3,710 (85.6) | 624 (14.4) |  | 1,018 (87.5) | 146 (12.5) |
| 4🡪5 | 4,253 (89.9) | 480 (10.1) |  | 1,465 (89.8) | 166 (10.2) |
| 5🡪6 | 4,183 (94.4) | 248 (5.6) |  | 1,758 (93.5) | 122 (6.5) |
| 6🡪7 | 4,021 (95.6) | 183 (4.4) |  | 2,048 (93.8) | 136 (6.2) |
| 7🡪8 | 3,410 (96.5) | 124 (3.5) |  | 2,088 (95.3) | 102 (4.7) |

All data are presented as No. (%).
